# Evaluation of multi-level barriers and facilitators in a large diabetic retinopathy screening program in federally qualified health centers: a qualitative study

**DOI:** 10.1186/s43058-021-00157-2

**Published:** 2021-05-22

**Authors:** Ana Bastos de Carvalho, S. Lee Ware, Tamara Belcher, Franceska Mehmeti, Eric B. Higgins, Rob Sprang, Cody Williams, Jamie L. Studts, Christina R. Studts

**Affiliations:** 1 Department of Ophthalmology and Visual Sciences, University of Kentucky, 110 Conn Terrace Ste 550, Lexington, KY 40508 USA; 2 Kentucky Telecare, University of Kentucky, Lexington, KY USA; 3 Division of Medical Oncology, Department of Medicine, University of Colorado Anschutz Medical Campus, Aurora, CO USA; 4 Cancer Prevention and Control Program, University of Colorado Cancer Center, Aurora, CO USA; 5 Department of Pediatrics, University of Colorado Anschutz Medical Campus, Aurora, CO USA

**Keywords:** Implementation, Barriers and facilitators, Multi-level factors, Federally Qualified Health Centers, Professionals, Primary care, Screening, Diabetes care

## Abstract

**Background:**

Recommended annual diabetic retinopathy (DR) screening for people with diabetes has low rates in the USA, especially in underserved populations. Telemedicine DR screening (TDRS) in primary care clinics could expand access and increase adherence. Despite this potential, studies have observed high variability in TDRS rates among clinics and over time, highlighting the need for implementation supports. Previous studies of determinants of TDRS focus on patients’ perspectives, with few studies targeting upstream multi-level barriers and facilitators. Addressing this gap, this qualitative study aimed to identify and evaluate multi-level perceived determinants of TDRS in Federally Qualified Health Centers (FQHCs), to inform the development of targeted implementation strategies.

**Methods:**

We developed a theory-based semi-structured interview tool based on the Consolidated Framework for Implementation Research (CFIR). We conducted 22 key informant interviews with professionals involved in TDRS (administrators, clinicians, staff). The interviews were audio-recorded and transcribed verbatim. Reported barriers and facilitators were organized into emergent themes and classified according to CFIR constructs. Constructs influencing TDRS implementation were rated for each study site and compared across sites by the investigators.

**Results:**

Professionals identified 21 main barriers and facilitators under twelve constructs of the five CFIR domains. Several identified themes were novel, whereas others corroborated previous findings in the literature (e.g., lack of time and human resources, presence of a champion). Of the 21 identified themes, 13 were classified under the CFIR’s Inner Setting domain, specifically under the constructs *Compatibility* and *Available Resources*. Themes under the Outer Setting domain (constructs *External Incentives* and *Cost*) were primarily perceived by administrators, whereas themes in other domains were perceived across all professional categories. Two Inner Setting (*Leadership Engagement*, *Goals and Feedback*) and two Process (*Champion*, *Engaging*) constructs were found to strongly distinguish sites with high versus low TDRS performance.

**Conclusions:**

This study classified barriers and facilitators to TDRS as perceived by administrators, clinicians, and staff in FQHCs, then identified CFIR constructs that distinguished high- and low-performance clinics. Implementation strategies such as academic detailing and collection and communication of program data and successes to leadership; engaging of stakeholders through involvement in implementation planning; and appointment of intervention champions may therefore improve TDRS implementation and sustainment in resource-constrained settings.

**Supplementary Information:**

The online version contains supplementary material available at 10.1186/s43058-021-00157-2.

Contributions to the literature

- Diabetic retinopathy is the leading cause of adult blindness in the USA, but is treatable with early detection and intervention. This is the first study to identify, classify, and prioritize multi-level barriers and facilitators of diabetic retinopathy screening via telemedicine in US safety-net clinics.
- Determinants associated with TDRS performance and perceived across all professional strata included leadership engagement, goal-setting, and performance feedback (Inner Setting domain), as well as intervention champions and staff education (Process domain).
- Strategies to improve TDRS implementation in the primary care setting could include academic detailing and collection and communication of program data and successes to leadership; engaging of stakeholders through involvement in implementation planning; and appointment of intervention champions.

## Introduction

Diabetic retinopathy (DR) is the leading cause of blindness in working age adults in the USA [1], and its timely detection and treatment reduce the risk of severe vision loss [2–4]. Success with early intervention is the basis for the DR screening guidelines of the American Academy of Ophthalmology, the American Diabetes Association, and the International Council of Ophthalmology, which recommend an annual (or in some cases biennial) eye exam or retinal photograph interpreted by an experienced reader [5–7]. Despite guidelines, as few as 18–33% of people in US communities with inequitable access to eye care (such as urban poor and rural communities) receive adequate diabetic retinopathy screening [8, 9]. Even among insured populations, as few as 15% of people with diabetes with no previously diagnosed DR receive adequate screening [10].

Telemedicine DR screening (TDRS) has been used for decades in national healthcare systems, such as the National Health System in the UK [11], as well as the Veterans Health Administration system in the USA [12, 13]. When screening achieves high rates of uptake and adherence to follow-up and treatment, diabetic eye disease can be dislodged as the leading cause of certifiable blindness among working age adults [11].

In the US, TDRS is increasingly being deployed to reach people with diabetes who otherwise may not adhere to recommended DR screenings [14]. With this technology, a non-mydriatic camera installed in a primary care clinic can acquire retina images during routine care visits of people with diabetes eligible for annual screening. Images are transmitted for interpretation by a remote reader, and a report is issued and sent to the requesting clinician with a recommendation for follow-up, tailored to the pathology found [15].

This approach eliminates several patient-level barriers associated with accessing in-person eye exams conducted by a specialist. Patient inconvenience, accessibility of the screening clinic (issues with transport and distance), time, and difficulty scheduling appointments have been identified as major barriers to DR screening [16–20]. For patients who attend primary care appointments, some barriers to screening may persist, such as financial concerns, competing health problems, and lack of symptoms [20], but many are eliminated when TDRS is offered during the appointment [21].

TDRS can have the most impact in low-resource populations, where factors such as access and patient education negatively impact DR screening rates [17, 22–25]. While the nature of TDRS alleviates patient-level barriers to screening, its integration into primary care clinical workflows is inconsistent, often suboptimal [26], with undefined best practices for implementation. Indeed, a significant proportion of people with diabetes treated in clinics equipped with TDRS technology remain unscreened [27, 28], suggesting that undocumented barriers may exist at a different level, for example, that of professionals involved.

To improve TDRS implementation in primary care clinics, more insight is needed regarding specific barriers and facilitators in this setting. Previous TDRS research has mainly focused on patients, with scarce data addressing barriers at the level of healthcare professionals (clinicians, nurses, staff) [29], organizations, or systems. Multi-level barriers and facilitators are particularly important in low-resource clinics where the decision to administer the exam is frequently made by staff rather than clinicians, and where priorities for resource allocation and patient care have to be carefully weighed.

The Consolidated Framework for Implementation Research (CFIR) [30] guided our conceptualization of multi-level factors influencing implementation of TDRS. The CFIR is a comprehensive, meta-theoretical framework of 39 constructs organized across five major domains theorized to influence implementation [31]. Importantly, these domains are multi-level and allow investigation of factors influencing implementation above the more commonly studied patient level. In this study, we addressed the five CFIR domains, as they relate to characteristics of clinicians, staff, and administrators involved in TDRS (Individuals Involved); the TDRS intervention (Intervention Characteristics); FQHCs (Inner Setting); the broader healthcare system (Outer Setting); and strategies for roll-out and operational integration (Process).

Implementation science approaches have rarely been applied in the field of ophthalmology [32–35], but offer powerful frameworks and methods to increase the public health impact of effective, yet under-utilized, eye care interventions. Thus, the goals of this study were to (1) use the CFIR to identify clinician-, staff-, organizational-, and systems-level barriers and facilitators to TDRS in low-resource primary care settings, and (2) identify influential CFIR constructs that explain implementation variability in this setting, operationalized by rates of TDRS delivery. We specifically targeted Federally Qualified Health Centers (FQHCs) serving urban poor and rural populations, and we conducted our data collection and analysis guided by the CFIR. The systematic identification of contextual factors associated with TDRS implementation is necessary to inform the next step in our program of research: the selection of tailored implementation strategies aimed at increasing the adoption and sustainment of TDRS in primary care clinics.

### Methods

The Standards for Reporting Qualitative Research (SRQR) checklist was used to guide reporting of methods and findings (Additional file 1). For complete methods, please see Additional file 2.

### Participants

Eligible clinics were FQHCs in an existing TDRS network described elsewhere [26], ensuring that all had some experience with the service. In selecting the sites for our study, we used maximum variation purposive sampling to obtain a diverse mix of clinic characteristics. We included a total of six clinics serving either rural (3 clinics) or urban (3 clinics) low-income communities. Additionally, we divided our clinical practices into three strata related to DR screening rates (high, medium, or low), and included 2 clinics from each stratus.

We applied maximum variation purposive sampling for the selection of individual participants as well, selecting from three types of professionals (i.e., clinicians, staff, and administrators) and including individuals with varying years in practice. The selected individuals received an emailed invitation to participate in the study. If a selected individual declined to participate, we invited another professional from the same clinic.

### Measures

A semi-structured interview guide was developed to allow participants to talk freely and volunteer rich information. A draft version was developed and pilot-tested. Modifications to the interview guide were made to improve question clarity and add newly identified lines of inquiry. The final semi-structured interview guide targeted constructs within all five CFIR domains (Additional file 3).

Sociodemographic characteristics of participants and clinics were collected using self-report via standard items, and included age, gender, race, ethnicity, professional role, and years in profession. Measured characteristics of clinics were provided by clinics’ administrators and included urban versus rural setting, and DR screening rate.

### Procedures

Face-to-face key informant interviews were conducted between August 2018 and March 2019. All interviews were audio-recorded and digital recordings were transcribed verbatim. The number of interviews completed was determined by data saturation: iterative coding was conducted as interviews progressed, with no new themes identified in the final two transcripts [36].

### Data coding and analysis

The investigative team developed a preliminary codebook for conventional content analysis through a process of discussion and refinement [37]. Briefly, trained researchers independently coded a sample of 3 transcripts using version I of the codebook and ATLAS.ti 8.4 software. The results of each coding were used to refine the codebook. Using version II of the codebook, the raters re-coded the first set of transcripts. When consensus was reached on a final codebook version and on coding of the sample transcripts, the coding process continued for the rest of the transcripts and a grid of emergent themes was developed. Two participants were invited for a member-checking process [38], following which the grid of themes was reviewed. Themes were then categorized into a CFIR-based matrix of relevant constructs and domains. For a complete method, see Additional file 2.

The identification of influential constructs followed methods described by Damschroder et al. [39]. Briefly, implementation effectiveness was characterized as high (2 sites), medium (2 sites), or low (2 sites), and a memo organized by CFIR construct was created for each site. Constructs were rated (as − 2, − 1, 0, + 1, + 2) based on the strength and valence of influence as perceived by key informants) through a deliberated consensus process for each memo (site). Ratings were compared across the two high and the two low implementation facilities to identify patterns in constructs distinguishing high and low implementation effectiveness. For a complete method, see Additional file 2.

## Results

### Participant characteristics

Of the 24 healthcare professionals contacted, 22 (92%) agreed to participate and completed the interview. The two who declined were primary care physicians (one male, one female).

Table 1 summarizes the characteristics of the professionals interviewed and clinic characteristics. The participating professionals varied widely in age and years of experience. Most were non-Hispanic White (*n* = 16/22), female (*n* = 21/22), and aged 40 or older (*n* = 15/22). In-profession experience ranged from 4 to 42 years, and the average number of people with diabetes seen weekly ranged from 6 to 75. Most participants worked in urban clinics (*n* = 15/22) and were distributed among clinics with low (*n* = 10/22), medium (*n* = 7/22), and high (*n* = 5/22) TDRS rates. Time of clinic use of TDRS services ranged between 1 and 4.5 years.

**Table 1:** Characteristics of health care professionals and clinics (*n* = 22)

Variable	***N***
Professional role	
Administrator	3
Clinician	9
Staff	10
Gender	
Male	1
Female	21
Age	
20–29 years	2
30–39 years	5
40–49 years	6
50–59 years	4
60+ years	5
Race/ethnicity	
Non-Hispanic White	16
Hispanic White	3
Non-Hispanic Black	2
Non-Hispanic Asian	1
Years in practice	
0–5	6
6–10	8
11–15	1
16–20	1
20+	6
Number of diabetic patients seen weekly by participant	
Don’t know	1
N/A (administrative role)	6
0–9	1
10–19	4
20–29	2
30–39	2
40–49	2
50+	4
Clinic setting	
Rural	7
Urban	15
Clinic DR screening rate	
Low (25th percentile)	10
Medium (26th–50th percentile)	7
High (50th–99th percentile)	5
Clinic experience with TDRS	
0–1 years	2
2–3 years	3
4+ years	1

*N/A* non-applicable, *DR* diabetic retinopathy, *TDRS* Telemedicine Diabetic Retinopathy Screening

### Qualitative themes by CFIR domain and professional role

**Fig. 1:**
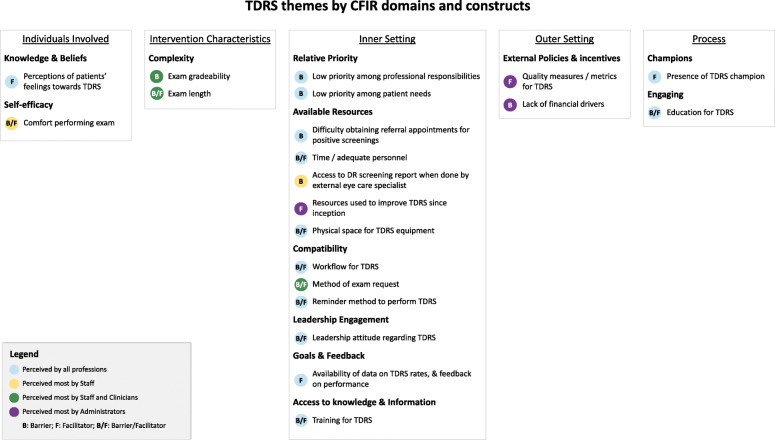
Telemedicine diabetic retinopathy screening themes by CFIR domains and constructs

Twenty-one themes emerged from interviews, which corresponded to twelve CFIR constructs and five domains (Fig. 1). Most themes described by participants were grouped under the domain Inner Setting (13 of 21 themes). Results are presented below, organized by CFIR domains and constructs, and by professional role (administrators, clinicians, staff) (Fig. 1).

#### Individuals involved

The first of two themes in this domain concerned perceptions of patients’ attitudes towards TDRS, consistent with the CFIR construct *Knowledge and Beliefs* (Fig. 1). Participants believed that the majority of patients were motivated by the convenience and ease of completing the diabetic eye exam in the primary care clinic:

*Low-income patients especially, like a lot of the migrant patients, they just, they feel comfortable here. […] I’ve found that it’s been very nice to like, at least get a screening test done and have that available here.* (Participant 9, clinician, urban clinic, low DR screening rate)

The second theme was participants’ comfort with the exam. Degree of comfort or confidence performing TDRS was perceived as either a barrier (for low degree) or facilitator (for high degree), and this theme aligned with the CFIR construct *Self-Efficacy* (Fig. 1). One staff supervisor noted how self-efficacy could influence screening, as staff with less TDRS experience and lower degree of confidence would only offer the exam to eligible patients if instructed by the clinician (frequently referred to as provider in the USA):

*Many of my more experienced CMAs they feel more comfortable to go ahead and do it themselves before provider will say it. And for some of those who are newer, they will do it only when the provider will remind them.* (Participant 4, staff supervisor, urban clinic, low DR screening rate)

#### Intervention characteristics

Two themes were associated with this domain: exam gradeability and exam length. These themes both reflect the CFIR construct *Complexity of the Intervention* (Fig. 1).

Many participants described gradeability of TDRS (whether the exam result is of sufficient quality to be interpreted) as a challenge. While the device provides user guidance and indicates whether the image obtained is adequate, acquiring a gradable picture required some user experience:

*As simple as the machine is and as automated as it is, there is still room for error. […] If you do it wrong you can get a bad picture, so that’s probably the biggest challenge.* (Participant 2, administrator, urban clinic, medium DR screening rate)

Similarly, the length of the exam was also perceived as a barrier by some clinicians and staff:

*Out of all the diabetic screenings, that one is the more time-consuming one.* (Participant 19, staff, urban clinic, low DR screening rate)

Several participants described exam length as a frequent reason for not offering TDRS to patients, especially in clinics in which TDRS is performed by the same staff who prepare patients for their clinical encounters. This perception was not universal, however. Other participants described needing only a few minutes to complete the exam, stating that the convenience and necessity of TDRS outweighed the potential disruption to clinic flow.

#### Inner setting

Most identified themes fell under this domain, and they clustered around 6 CFIR constructs: (1) *Relative Priority*, (2) *Available Resources*, (3) *Compatibility*, (4) *Leadership Engagement*, (5) *Goals and Feedback*, and (6) *Access to Knowledge and Information* (Fig. 1).

##### Relative priority

Many clinicians and staff described the priority of TDRS as low relative to other exams necessary for people with diabetes, particularly for those with multiple pathologies. This low relative priority was clear in several participants’ descriptions of screening decisions:

*They come in with a million problems […], and most of the time you just have to prioritize which are more important, and they always have a lot of acute problems that need attention and so things like screening sometimes just get dropped on the bottom.* (Participant 8, clinician, urban clinic, low DR screening rate)

Several participants mentioned that changes in other clinic workflows and systems that coincided with TDRS implementation made it more challenging to adopt the exam, suggesting that low relative priority was given to TDRS implementation:

*We’ve been using these EMR forms for a little over six months, and we’ve changed something a couple weeks ago. And people are now like, ‘another change…’, you know, so they’ve had to adapt to that.* (Participant 1, administrator, urban clinic, low DR screening rate)

##### Available resources

Professionals described lack of resources and time as a barrier to TDRS. In most participating clinics, TDRS was performed by medical assistants (healthcare workers trained for the role of clinicians in tasks such as taking medical histories, recording vital signs, and other administrative and clinical tasks). This was in addition to their other responsibilities (such as those detailed above), and this lack of dedicated personnel to perform TDRS was perceived as an impediment:

*It comes down to the time piece. If it just feels overwhelming, if I’m overwhelmed and they’re overwhelmed […] and then there’s this diabetic eye exam, that may fall through the cracks.* (Participant 9, clinician, urban clinic, low DR screening rate)

The existence of dedicated staff for TDRS was considered a potential facilitator by some professionals:

*In my wish list, the nurse I would have just for diabetic patients, for education, and this nurse will be […] bringing patients and doing this [TDRS exam] herself and making sure of quality.* (Participant 4, staff supervisor, urban clinic, low DR screening rate)

Availability of physical space was another key theme. This TDRS system required a dark room and space for the desktop camera, and participants considered existence of adequate space as a facilitator. Similarly, some clinics allocated resources to improving TDRS workflow after an initial trial period (e.g., the camera was moved to a more convenient location; the exam room was darkened with curtains). Consistently, difficulties finding appropriate space for the camera posed challenges for implementation efforts.

Resource scarcity was also described as influencing communication between clinics and external clinicians. Specifically, participants attributed issues with retrieving reports of eye exams performed elsewhere to a lack of staff. Difficulties documenting whether screening had been performed elsewhere acted as a barrier to TDRS, since most professionals would not order it if patients mentioned having an eye exam in the past 12 months (even if this was not documented):

*The provider has said I’m not going to send them for an eye screen if they’re telling me they’ve already had one, but then maybe their health reminder is not satisfied because we can’t get the records.* (Participant 7, staff, urban clinic, medium DR screening rate)

Some participants also reported difficulties accessing an eye care specialist for patients with referable pathology found in TDRS, explaining that this translated into frustration with providing screening:

*Once they have an abnormal scan […] I’m going to send them to an eye doctor anyway. So the issue I have is getting them to go back to the eye doctor […] because that’s another appointment and it's outside.* (Participant 22, clinician, urban clinic, medium DR screening rate)

##### Compatibility

Compatibility of TDRS with clinic systems and workflows was identified as both a facilitator and a barrier. The reminder for TDRS was seen as a facilitator when it was considered reliable, which happened mostly when the reminder was activated by a staff who “scrubbed” charts (i.e., manually reviewed the chart prior to the appointment):

*We do have scrub sheets here that we are able to look at, that they let us know if certain things need to be done at the time of their visit, so if “eye exam” is marked on there, we can try to go ahead and perform that.* (Participant 16, staff, rural clinic, high DR screening rate)

However, one administrator mentioned that electronic medical records (EMR) reminders were not trusted, as they were automated and lacked integration, which left clinicians without a reliable reminder for TDRS.

All clinics in the study used the standing order method of request for TDRS. With this method, the exam order was conditioned upon the occurrence of certain criteria—a person with diabetes without a diabetic eye exam in the past 12 months—and patients who met these criteria could receive the exam without the need for a physician order. One administrator noted that, regardless of a standing order, when TDRS was specifically requested by a clinician (not left to the discretion of the staff to perform), exam rates increased. Thus, request by clinician was seen as a facilitator, but one that was not always compatible with usual clinic procedures and flow.

In a parallel theme of compatibility with existing workflows, lack of integration of exam reports into the existing EMR system was an identified barrier.

*For something like a retinal image that’s read outside and we get a scanned document back in, [the EMR] doesn’t automatically populate those fields. And so we’ve done a lot of work to try to develop an abstracting system, so that when we get those [TDRS] reports back, not only are we scanning those in for the provider to review and sign off, but we’re also entering the completion date into a reportable field.* (Participant 11, administrator, rural clinics, medium and high DR screening rates)

##### Leadership engagement and goals and feedback

These CFIR constructs tended to appear jointly in interviews. Most participants perceived engagement and feedback from leadership as facilitators, explaining that clinic directors and administrators had high engagement in this exam:

*If you are falling way low you get, you know, emails from the medical director that you need to get your numbers of your diabetics, and your diabetic retinopathy scans done.* (Participant 10, clinician, rural clinic, medium DR screening rate)

When clinicians were described as leaders, their engagement was identified as either a barrier or a facilitator, depending on whether they reinforced and supported staff in initiating TDRS:

*The focus is more on controlling the hemoglobin A1C, so I think that’s probably is what people look at, at the physician level, and I think what the physician says, kind of flows down to the support staff.* (Participant 1, administrator, urban clinic, low DR screening rate)

*It all ends up being in our, like the providers’ hands, basically more or less. The staff know that they are supposed to do [the TDRS exam], and periodically they are reinforced to.* (Participant 8, clinician, urban clinic, low DR screening rate)

Several participants explained that sharing data regarding TDRS rates would be a valuable way of providing feedback and increasing adherence to the exam:

*Data is helpful when you go back and review […]. I can send this out, the graph out showing how many we’re doing a month. I can report that out to our providers and show them where we are* (Participant 6, staff, urban clinic, medium DR screening rate).

##### Access to knowledge and information

While TDRS is considered easy to perform by even non-eye care professionals, many staff felt like their training had been insufficient:

*We weren’t really sure what were the appropriate images, what was acceptable, what wasn’t. Again, it seemed like we had to kind of learn more of that on our own, so I do think there could have been a little bit more training.* (Participant 6, staff, urban clinic, medium DR screening rate)

Participants also believed that the training plan—one initial stand-alone session without refreshers—was inadequate in clinics with high personnel turnover. This left new staff either untrained or trained by a colleague, which was felt to be less adequate than the training provided initially by the expert trainer.

Outer setting

Within this domain, the primary identified theme was the lack of a financial driver for TDRS, despite its relatively low cost, consistent with the CFIR construct *External Policies and Incentives* (Fig. 1). Administrators described complexities in obtaining reimbursement for TDRS, which typically resulted in clinics not billing for performed screenings. Additionally, while Medicare and Medicaid include DR exam as a quality measure, this potential facilitator was described as having minimal influence, due to low economic incentives for attaining these metrics.

There’s not a pressing financial driver for these things.[…] There’s a lot of talk about in the future getting paid more for quality. Right now the way the finances are I’m not aware that there’s a driving thing (Participant 2, administrator, urban clinic, medium DR screening rate)*.*

#### Process

Themes in this domain were consistent with the CFIR constructs *Engaging* and *Champion* (Fig. 1).

##### Engaging

This CFIR construct refers to efforts to attract and involve individuals in the use of an intervention through activities such as marketing, training, or education [31]. A lack of engagement was noted by some clinicians and staff, describing uncertainty with the sensitivity of the technology. Participants suggested that access to education about the importance of DR screenings could work as a facilitating tool:

*They need to feel empowered to make that decision and why they’re doing it. It’s not just another checked box on their list, that “They’re diabetic, I have to do this”. […]. If we had more education for them, for them the support staff, it may become, it may help us bump that priority side.* (Participant 3*,* staff supervisor, urban clinic, low DR screening rate)

##### Champion

While most clinics in the study did not have a recognized champion, participants agreed that such an agent would likely promote use of TDRS. Within the few clinics that did have a champion, all participants identified that individual as an influential facilitator:

*She’s the one that makes sure that they’re done to the best of their abilities and that people are doing [the TDRS exams] right. […] She made it her mission and went above and beyond for it.* (Participant 22, clinician, urban clinic, medium DR screening rate)

### Perceptions of barriers and facilitators by professional role

We found that the majority of themes were identified across all professional roles (Fig. 1). When analyzed by CFIR domain, themes falling into the Outer Setting (construct *External Policies and Incentives*) were mostly perceived by administrators. Conversely, themes aligned with the domain Intervention Characteristics (construct *Complexity*) were emphasized by clinicians and staff. Staff were also more likely to refer to issues with comfort performing the exam (Individuals Involved domain, construct *Self-efficacy*), as well as to difficulties accessing DRS reports for patients who were screened by an eye care clinician (Inner Setting domain, construct *Available resources*).

### Identification of constructs distinguishing sites with low versus high TDRS implementation effectiveness

We assessed the 12 CFIR constructs that were consistent with the identified themes and rated each as having positive or negative influence on TDRS performance (Table 2). We found that four constructs were perceived as strongly distinguishing low from high implementation effectiveness, defined by rates of TDRS delivery. Two were related to Inner Setting (*Leadership Engagement,* and *Goals and Feedback*), and two were related to Process (*Engaging* and *Champion)*. Two additional constructs weakly distinguished sites (*Relative Priority* and *Available Resources)*, and four were non-distinguishing (*Complexity*, *Access to Knowledge and Information*, *Compatibility*, and *External Policies and Incentives*). We considered the constructs *Knowledge and Beliefs* and *Self-Efficacy* (Individual domain) as not applicable, because the focus of our ratings was not on individual-level behavior change but rather on clinic-level characteristics. Additional file 4 provides quotes and memo details on how each construct manifested in the study sites.

**Table 2:** Ratings assigned to CFIR construct by site

Implementation effectiveness	Low		Medium		High		
Site ID	102	104	105	106	101	103	
**I. Individuals involved**							
Knowledge and beliefs	1	1	1	1	1	1	
Self-efficacy	N/A	N/A	N/A	N/A	N/A	N/A	
**I. Intervention characteristics**							
Complexity	0	− 2	− 1	2	1	0	
**III. Inner setting**							
Relative priority	− 1	− 1	− 1	0	1	0	*
Available resources	− 1	− 1	(mixed) 0	1	1	(mixed) 0	*
Compatibility	0	0	1	1	1	1	
Leadership engagement	− 1	0	1	1	2	2	**
Goals and feedback	− 1	0	1	1	2	2	**
Access to knowledge and information	0	− 1	− 1	− 1	1	0	
**IV. Outer setting**							
External policies and incentives	1	1	0	1	2	2	
**V. Process**							
Engaging	− 1	− 1	Missing	(mixed) 0	Missing	2	**
Champions	− 1	− 1	Missing	2	2	Missing	**

**Construct strongly distinguishes between low and high implementation effectiveness

*Construct weakly distinguishes between low and high implementation effectiveness

## Discussion

Despite the potential for TDRS to increase DR screening rates in primary care settings, its adoption is often suboptimal. This qualitative study (1) identified perspectives of staff, clinicians, and administrators in FQHCs regarding barriers and facilitators to TDRS, (2) determined differences in perceptions of barriers and facilitators by professional category, and (3) prioritized CFIR constructs based on their perceived influence on implementation effectiveness, with the goal of informing implementation and sustainment strategies towards improved patient outcomes.

Our findings that constructs perceived to distinguish high versus low implementation sites cluster in the Inner Setting and Process domains of CFIR are consistent with implementation research on other evidence-based practices and interventions [39–42] and indicate influential targets for implementation strategies.

### Common barriers to TDRS implementation

Resource constrains were observed to affect exam performance, accessing prior results, making referrals, and tracking screenings over time. This finding is unsurprising given previous evidence that resource scarcity hinders sustained program implementation of evidence-based interventions in general in low-resource environments [43]. Specific to TDRS, challenges in obtaining timely referrals and treatment for patients with pathology found through the screening have previously been noted as barriers [44], consistent with our findings.

Poor integration of TDRS with existing EMR systems compounds the perceived lack of resources, as it requires extra time and effort in an already strained environment. Technological challenges perceived as increasing workload are a common barrier to telemedicine uptake in general [45]. Software solutions that integrate TDRS devices and outputs into EMRs are necessary but may be costly. Identification of less expensive strategies to target this barrier, such as additional training or interventions that support self-efficacy towards new technology, may be needed.

Low relative priority is a common cause of poor implementation of a wide range of innovations in primary care [46], and this was the case for TDRS as well. Combined with the stresses of inadequate resources dedicated to TDRS, low relative priority and low motivation reported by clinicians and staff may be important targets for intervention.

In addition to the Inner Setting themes discussed above, professionals identified exam complexity as a barrier to consistent use of TDRS. In most clinics, workflow was not formally modified to accommodate this new test, which likely exacerbated these perceptions. Instead, workaround strategies (informal temporary practices for handling exceptions to normal workflow) [47] were frequently used, and staff were assigned TDRS as an additional task to squeeze into their day. While workarounds are commonly used in medical settings [48], and specifically in TDRS services [49], they can increase medical errors [50] and create further load in already resource-constrained clinics [49]. Therefore, while sometimes necessary, workarounds should be minimal, temporary, and replaced with formal workflow adjustments [50] as soon as possible.

At the level of individuals, several staff and clinicians expressed low self-efficacy in performing TDRS, linked with perceptions that their training had been inadequate. Training of personnel is a basic and necessary implementation strategy [43], but alone it is generally insufficient [51]. Consideration of enhanced training modalities and supports is warranted to target this barrier and may include strategies suggested by key informants, such as more training at inception of TDRS, continued training sessions going forward, and easily accessible videos or manuals for troubleshooting.

### Distinguishing constructs as primary targets for implementation strategies

The process of selecting targets for generalizable implementation strategies can be fraught with difficulty, due to variation in barriers and to challenges matching strategies to targets for change. One pragmatic approach is to prioritize contextual factors (or constructs) that distinguish between high- and low-implementing sites in observational analyses. Targeting such constructs may lead to more effective implementation and sustainment of TDRS.

In this study, we consistently found that in the Inner Setting domain, the constructs *Leadership Engagement* and *Goals and Feedback* strongly distinguished high and low implementation sites. Indeed, engaged and consistent leadership in primary care can have a positive impact on adoption of specific practices, whereas low engagement by leaders can present as a barrier [46]. Stakeholder feedback highlighted the importance of engaged leaders communicating with clinicians and staff about TDRS and providing frequent and effective reinforcement and feedback based on program data.

Also in the Inner Setting domain, the constructs *Relative Priority* and *Available Resources*, which have also been shown to influence implementation of evidence-based practices across settings [39, 52], similarly distinguished between high- and low-performing sites, though less strongly. While resources were constrained at all sites, clinics categorized as high- and medium-performing sites with respect to TDRS rates were mostly able to overcome resource scarcity by reallocating personnel and space, adapting the EMR system, and redesigning workflows. These adjustments seemed to be motivated by higher priority of TDRS in these sites, where the exam was seen as equally important to other diabetic screenings. Further, by anticipating the inevitable resource burdens [53] of a new exam, these measures may have contributed to reduction in the perceived complexity of the intervention [54], with more seamless adoption and sustainment.

Our findings regarding these four Inner Setting constructs suggest that implementation of TDRS in low-resource settings may benefit from interventions targeting leadership engagement (such as collection and communication of program data and successes; academic detailing) and appropriate planning of required resources (reallocation of space, funds, and personnel time). Targeting such factors may lead to a heightened perception of relative priority by stakeholders and a stronger implementation climate [55].

In the Process domain, the constructs *Engaging* and *Champion* strongly distinguished implementation effectiveness. The value of upfront planning for implementation of new technology or telemedicine services, especially in resource-constrained settings, cannot be overstated [56]. Sites with more thorough implementation planning, which included engaging of stakeholders (i.e., staff and clinicians who would be directly involved in TDRS), were more likely to have higher implementation effectiveness, consistent with studies in other fields [57]. Further, sites with TDRS champions had more active promotion of TDRS, quicker resource mobilization, and more efficient feedback about TDRS to health professionals. These Process factors are critical for implementing change in primary care [58], and affirm the importance of implementation strategies for TDRS in FQHCs that engage stakeholders (e.g., education, involving stakeholders in planning, tailoring TDRS to prospective barriers, and designating a project champion).

### Limitations

While our analysis identified perceived factors that distinguished between high and low implementation effectiveness, our study design does not permit us to determine which of these are most important for TDRS implementation, nor how they are associated with characteristics of each profession. As with any qualitative study, social desirability bias is a possibility; however, participants seemed frank and genuine in relaying their experiences with TDRS in their clinics. Our sampling strategy precludes generalizability of results beyond the participants in this study; however, our purposeful selection of clinics and key informants ensured a range of experiences and perceptions among study participants. In one of six sites, we were not able to attribute ratings to the constructs *Engaging* and *Champion*. This may decrease the strength of the evidence that these constructs strongly distinguish sites. Nonetheless, the pattern of ratings across low, medium, and high sites for these constructs is striking and may suggest a dose-response relative to site performance.

We plan to address the inherent limitations of qualitative research in the next phase of our research, in which quantitative survey methods will be used to investigate the prevalence and relative importance of themes identified in this study among a representative sample of health professionals and practice sites in our network.

Despite these limitations, this study offers insight into perceived challenges for adoption of TDRS in primary care clinics serving rural and urban poor populations in the USA, where the telemedicine approach to DR screening could have its greatest impact. This is, to our knowledge, the first evaluation of the relative influence of multi-level barriers and facilitators—categorized using CFIR—on the implementation of TDRS. We examined the views of a diverse group of health care professionals involved in healthcare delivery to people with diabetes within clinics that have had experience with TDRS. Participating clinics included rural and urban sites with varying years of experience in using TDRS, as well as varying rates of screening. Further, the use of theory for collection and analysis of our qualitative data enhanced the scientific rigor of this research [59].

## Conclusions

Our results highlight the interactive and multi-level nature of factors influencing the implementation of TDRS in primary care. While patient attitudes and perceptions of TDRS are important, barriers and facilitators at the clinician, clinic, and systems levels must be addressed to improve its adoption and sustainment. Classification of emergent themes into CFIR domains and rating of constructs distinguishing high- and low-performing sites provided an actionable organization of results that will facilitate the development of targeted implementation strategies to improve the use of TDRS in FQHCs, and in other settings with comparable characteristics. Similar methods can be used to gain insight into implementation of other telemedicine-based interventions in primary care centers, specifically in clinics providing for underserved populations.

## Supplementary Information

**Additional file 1.** Standards for Reporting Qualitative Research checklist.

**Additional file 2.** Supplementary methods.

**Additional file 3.** Complete interview structure.

**Additional file 4.** Manifestation of CFIR constructs.

## Data Availability

The datasets generated and/or analyzed during the current study are not publicly available due to participant confidentiality considerations. Aggregate data are available from the corresponding author on reasonable request.

## Abbreviations

DR: Diabetic retinopathy
TDRS: Telemedicine Diabetic Retinopathy Screening
FQHC: Federally Qualified Health Center
CFIR: Consolidated Framework for Implementation Research
EMR: Electronic medical records
