# Supplementary material for: Evaluation of multi-level barriers and facilitators in a large diabetic retinopathy screening program in federally qualified health centers: a qualitative study
Source: Implement Sci Commun. 2021 May 22;2:54. doi: 10.1186/s43058-021-00157-2 (PMC8141191; doi:10.1186/s43058-021-00157-2)
Supplement: Supplementary file 2 — Additional file 2. Supplementary methods. [file 43058_2021_157_MOESM2_ESM.docx]

Additional File 2

Complete Methods

**Participants**

Eligible clinics were part of an existing TDRS network, ensuring that all had some experience with TDRS. In selecting the sites for our study, we used maximum variation purposeful sampling to obtain a diverse mix of clinic characteristics. Clinics serving either rural (3 clinics) or urban (3 clinics) poor communities were included, so as to collect data in both types of populations and environments. Rurality was defined by the US classification system whereby each community was at the non-micropolitan level (less than 9,999) or micropolitan level (10,000 to 49,999)(1). Using these criteria, we selected 3 urban and 3 rural clinics from our network. Additionally, we divided our clinical practices into three strata related to DR screening rates (high, medium, or low), and included 2 clinics from each stratus. Rates were calculated using known DR screening rates in the TDRS network for the calendar year of 2018. Clinics in the 25^th^ percentile for screening rates (DR screening rate <23%) were considered low screeners; clinics in the 26^th^-50^th^ (DR screening rate 23-44%) percentile were considered medium screeners; and clinics above the 50^th^ percentile (DR screening rate >44%) were deemed high screeners.

**Semi-structured interview guide**

A semi-structured interview guide was developed to allow participants to talk freely and volunteer rich information. A draft version was developed and pilot-tested with one volunteer clinician and one volunteer staff (a nurse practitioner). Modifications to the interview guide were made to improve question clarity and add newly identified lines of inquiry. The final semi-structured interview guide targeted constructs within all five domains of the Consolidated Framework for Implementation Research (CFIR). We chose to use this framework because the constructs within each CFIR domain can be directly operationalized as interview questions (2), and because the framework can be integrated throughout the research process (design, data collection, coding, analysis).The interview guide started with an introductory section and warm-up questions, followed by questions covering the integration of TDRS in the clinic (exam request, workflow, training). Next, perceptions about TDRS in participants’ clinics and about clinic characteristics were addressed (e.g., typical patient workflow, structure of employees, culture, endorsement by leaders, changes to workflow and integration, champions, goals for TDRS, high priority activities). Finally, participants were asked to reflect on additional perceived barriers and facilitators, as well as to provide suggestions on improvement of TDRS implementation. Auxiliary questions were added for clinicians regarding their perceptions of DR screening rates in their patients, as well as their alignment with expected rates according to internal or external goals and guidelines.

**Procedures**

Face-to-face key informant interviews were conducted between August 2018 and March 2019 by one of two trained interviewers (ABC and TB), one a practicing ophthalmologist and the other a nurse in rural practice. Mean interview duration was 40 minutes (range 19–55 minutes). All interviews were audio-recorded and digital recordings were transcribed verbatim. Identifying information was removed from transcripts before analysis. The number of interviews completed was determined by data saturation (see below) (3). Sociodemographic characteristics were collected at the end of the interview for 12 participants, and via email for 10 participants. Participants received a fifty-dollar compensation for their time.

**Data coding and analysis**

The investigative team developed a preliminary codebook for directed content analysis through a process of discussion and refinement (4). Trained researchers (ABC, TB and FM) each independently coded a sample of 3 transcripts using version I of the codebook using ATLAS.ti 8.4 software. The results of each coding were compared to identify concordant and discordant coding, then used to refine the codebook. Using version II of the codebook, the raters re-coded the first set of transcripts. Discrepancies were discussed until consensus was reached on a final codebook version and on coding of the sample transcripts. The coding process then continued for the rest of the transcripts. All transcripts were coded by two analysts (ABC and TB, or FM and ABC) who compared their codes transcript by transcript. As coding progressed, the investigative team developed and reached agreement on a grid of emergent themes. Once initial coding was completed, two participants were invited to participate in a member-checking process to determine whether additional data collection was necessary and to ensure valid inferences were made through coding procedures (5). Following member checking, the investigative team reviewed and refined the grid of themes. The themes were categorized by consensus into a CFIR-based matrix of relevant constructs and domains. Each theme was classified as barrier, facilitator, or dichotomous (both barrier and facilitator).

**Identification of influential constructs**

Identification of influential constructs explaining variation in TDRS implementation was done as previously described (6). We firstly characterized implementation effectiveness in our sites. Because our intervention includes only one component (TDRS exam, measured as TDRS rates) we used TDRS rates to characterize implementation effectiveness. Two clinics were characterized as having high implementation effectiveness (high screeners, see above), two as medium (medium screeners), and two as low (low screeners). For each facility, individual interviews were consolidated into case memos (a pair of analysts wrote a case memo for each site, organized by CFIR construct). In the memos, each construct had a summary statement and supporting quotes. The six resulting memos were then rated independently by two analysts for each construct (as -2, -1, 0, +1, +2) through a deliberated consensus process. Ratings reflected 1) positive or negative influence, and 2) strength, of each construct in each facility, and represented a consolidated assessment across all interviews. Ratings were intended to capture the extent to which the construct was perceived to affect the implementation process. The aggregation of data in each construct was assigned a valence between − 2 to + 2, representing the perceived direction (positive or negative) and perceived strength (− 2 to + 2) of the construct on implementation. Constructs without perceived indication of an effect were assigned the valence zero. Mixed effects were rated as “(mixed)”. Ratings were then compared for each construct across sites to help ensure consistency. Disagreements were addressed through consensus discussions. For analysis of the ratings, we focused on discerning patterns across the two high and the two low implementation facilities, which allowed us to identify patterns in construct ratings that distinguished between high and low implementation effectiveness. Constructs were characterized as: strongly or weakly distinguishing low versus high implementation facilities, or not distinguishing. We used an approach similar to Damschroder et al (6) to determine strongly or weakly distinguishing constructs. Constructs were considered strongly distinguishing when the difference between rating averages for high and low implementation sites was at least 2.5. (e.g. ratings for high implementation sites +2, +2; Ratings for low implementation sites-1, 0; difference of averages: +2-(-0.5) = 2.5). Constructs were considered weakly distinguishing when the difference between rating averages for high and low implementation sites was at least 1.5. These differences between averages are similar to those that emerge from analysis of the original methodology paper by Damschroder et al. Further, when the difference between rating averages was 1.5 or less and ratings for sites in the same category were discordant by at least two points (e.g. ratings for low implementation sites for a particular construct were -2 and 0), the evidence was considered insufficient to assert that the construct was distinguishing, and the construct was considered not distinguishing.

**References**

1. United States Census Bureau: Geocoder: <https://geocoding.geo.census.gov/>. [

2. CFIR Research Team: CFIR technical assistance website: <https://cfirguide.org>; [

3. Francis JJ, Johnston M, Robertson C, Glidewell L, Entwistle V, Eccles MP, et al. What is an adequate sample size? Operationalising data saturation for theory-based interview studies. Psychology & health. 2010;25(10):1229-45.

4. Hsieh HF, Shannon SE. Three approaches to qualitative content analysis. Qualitative health research. 2005;15(9):1277-88.

5. Fielding NF. Linking data. Newbury Park, CA: Sage; 1998.

6. Damschroder LJ, Lowery JC. Evaluation of a large-scale weight management program using the consolidated framework for implementation research (CFIR). Implementation science : IS. 2013;8:51.
