# Supplementary material for: Evaluation of multi-level barriers and facilitators in a large diabetic retinopathy screening program in federally qualified health centers: a qualitative study
Source: Implement Sci Commun. 2021 May 22;2:54. doi: 10.1186/s43058-021-00157-2 (PMC8141191; doi:10.1186/s43058-021-00157-2)
Supplement: Supplementary file 3 — Additional file 3. Complete interview structure. [file 43058_2021_157_MOESM3_ESM.docx]

**Additional File 3**

**Question Guide for Key Informant Interview**

1. Introduction

- Interviewer introduce themselves
- Explanation of background of the study
- Information about how the interview will be conducted, estimated length, audio recording authorization request.
- Request participant to describe their profession, years of experience, years working in the clinic, number of patients seen with diabetes each month

1. Workflow for a patient with diabetes that arrives in clinic

Prompts

- Reminder method for an eye exam for diabetic patients
- TDRS request process
- Patients feelings and opinions about TDRS process

1. TDRS integration into current clinic processes and workflows

Prompts

- General structure of employees in the clinic (administrative, clinicians, staff, coordinators, nurses, dietitians, referral specialists)
- Location of the camera/who executes the exam
- Electronic health records
- Process of getting TDRS performed
- Training of staff (initial/refreshers)
- Ease of use of camera and exam acquisition
- Referral process for positive screenings
- Follow-up process for patients with referrals

1. Has the TDRS process always worked this way or were changes made

Prompts

- Change in location
- Change in equipment
- Change in workflow
- Change in request format

1. Efforts to improve the use of TDRS

Prompts

- Goals for TDRS set in the organization
- Feedback from leadership
- Quality measures that involve DR screening
- Endorsement/barriers for TDRS from leaders
- Billing/reimbursement

1. Relative priority of TDRS in the clinic

Prompts

- High-priority activities in the clinic
- Pressure to perform higher priority activities

1. Champion for TDRS

Prompts

- Formally appointed or informal role
- How does champion facilitate use of TDRS

1. Other factors that affect the use of TDRS in the clinic
2. What suggestions do you have to improve the process and use of TDRS
