# Supplementary material for: Evaluation of multi-level barriers and facilitators in a large diabetic retinopathy screening program in federally qualified health centers: a qualitative study
Source: Implement Sci Commun. 2021 May 22;2:54. doi: 10.1186/s43058-021-00157-2 (PMC8141191; doi:10.1186/s43058-021-00157-2)
Supplement: Supplementary file 4 — Additional file 4. Manifestation of CFIR constructs. [file 43058_2021_157_MOESM4_ESM.docx]

**Additional File 4**

**Manifestation of constructs that differentiated between high and low implementation clinics**

|  |  | **Low** | | **Medium** | | **High** | |
| --- | --- | --- | --- | --- | --- | --- | --- |
| **INNER SETTING** | |  | |  | |  | |
|  | **CFIR Ratings:** | **-1** | **-1** | **-1** | **0** | **+1** | **0** |
|  | RELATIVE PRIORITY | Several professionals in all clinics considered TDRS as a lower priority exam than other exams in diabetics, as diabetic patients frequently presented with severe multiple co-morbidities that needed management, as well as urgent issues, such as uncontrolled glycemias (blood sugar), chest pain, or infections. | | | | | |
|  |  | *I do think that that becomes an issue for us just you know transportation, food, housing, it’s just, you’ve got so much to deal with, that you start thinking, oh well you’re not complaining of any visual stuff, maybe we can check on this next month. But I got to figure out where you’re going to live and where you’re going to get your next meal from, so those things kind of overtake the checklist.* [Administrator, 102]  Additionally, TDRS had lower priority at both clinics, relative to other recent initiatives. Namely, both sites had a major change in the EMR system, which was time- and effort-consuming for all administrators, providers and staff, leaving the newly implemented TDRS as a lower priority activity:  *We’ve been using these EMR forms for a little over six months, and we’ve changed something a couple weeks ago. And people are now like, another change, you know, so they’ve had to adapt to that. [*Administrator, 104] | | *I think there’s lots of competition. So, the patient feels crummy, do we want to put them through this today, do we want to just … address what brought them in and let go home and go back to bed…We see a lot of mental health so maybe they’re like super, super depressed.  And again like you’ve just spent 30 minutes with the patient crying in your office and so “let’s do this retinal exam” just feels not good.* [Administrator, 105]  The administrator of one of the sites also mentioned that during certain periods of the year providers felt pressure to reach their goal number of patient visits. This could lead to shorter visits and overlooking TDRS, which was considered a time-consuming exam:  *In terms of provider pressure, of you know seasonality, it’s the time of year when volume needs to increase…that’s when I might shorten this, skip this thing*  [Administrator, 105] | | At one clinic, despite the perception that diabetic patients have multiple competing health issues, participants felt like TDRS still had high priority and was an important component of clinical care:  *Most diabetics also have heart disease, they also have hypertension, they also have an acute problem on top of their heart disease, hypertension, hyperlipidemia, diabetes that they came in for, so of course the acute problem is top in priority.  Diabetes care… unless there’s an acute issue… I would say they’re all equal in terms of…eye examination, foot care, kidneys…they’re all important. When you come in today, you’re diabetic, and you’re behind on your eye examination, your foot examination, your micro albumin, that’s all going to get done.*  *[*Administrator, 103] | |
|  | **CIFR Ratings:** | **-1** | **-1** | **0 (mixed)** | **+1** | **+1** | **0 (mixed)** |
|  | AVAILABLE RESOURCES | Other than space, additional resources (e.g., staff time, funding) were not provided to clinics for TDRS. Thus, resources were constrained at all of the clinics in our study. However, each clinic handled this relative lack of resources, and its consequences for TDRS, differently. | | | | | |
|  |  | At one site, participants noted the recent addition of scribes to the practice and the presence of referral staff, as a positive factor that would likely increase providers time henceforth for other tasks, such as TDRS. However, these professionals were not available until recently, and their absence since the implementation of TDRS had been noted. Further, participants noted a relative lack of other human resources like support staff (such as MAs or nurses) dedicated to closing the loop on TDRS (identifying eligible patients, ensuring it was performed, referring positive screens and ensuring follow-up was held)), whose absence increased the burden of time needed to complete TDRS:  *If we would have nurse who would, is in my wish list, the nurse I would have just for diabetic patients for education and if this nurse will be one who will be bringing patients and doing this herself and making sure of quality, and then make sure that this report got back to the clinic and it got back to the patient and patients really understand that what they’re dealing with.* [Staff supervisor, 104]  At another site, there was no dedicated space for the TDRS equipment, which acted as a major barrier:  *Space is very limited and that’s part of the problem for them, and that [the provider] is like “Hurry up, get the patient in the room”. And the support staff are trying to get them in the room and they’re doing somebody, and they’re weighing, and they’re doing blood pressure, and there’s something in the room, and that’s a problem. [*Staff supervisor, 102]  Further, this clinic was struggling with lack of general medical assistants (staff) who were more involved with TDRS training and execution, as several had quit in the past few months:  *One of the people who left, he was actually going in just double checking weekly so just to keep an eye on [TDRS] a little bit more. We haven’t been able to do that as much. But I think that was very beneficial as well. But we haven’t been able to do those much since he left. Unfortunately we had two people [trainers for TDRS], three people, and two of them are gone. So now we just have one.*  [Staff supervisor, 102] | | At one site, participants identified having resources like scrub nurses that flag eligible patients for TDRS, and no significant difficulty accessing DRS reports when the exam is done at an external eye care provider. However, there was a relative lack of staff to work up patients and perform TDRS, which reflected as participants referring to lack of time to complete the exam:  *It’s really busy there and so… just the pace. Like this is one of the in-office procedures that we do that takes the most time and so if they were going to skip something. …Not like they would intentionally skip it but it’s likely for them to feel like “I don’t have time for that today, I’ll just do that next time, that can happen next time.*  [Administrator, 105]  One site had a multitude of resources such as scrub personnel that flag eligible patients for TDRS, standing orders for TDRS, a diabetes nurse educator, and an internal QI committee. This site was also unusual in that it had a dedicated person executing TDRS, which worked as a facilitator, as TDRS didn’t take time from the MAs’ clinical duties. However, stakeholders did feel like integration with the EMR was deficient, and that getting reports from external DRS was cumbersome and difficult. | | In one site, participants felt like there was a relative lack of resources but that it could be overcome in most instances. Providers and staff had a different perception about the time needed for TDRS, than participants at other sites, such as: *“Oh goodness, it don’t take no time, maybe 2 minutes, 3 minutes.  I mean, it doesn’t take long at all”* [Staff, 101]  On the second site, staff complained of lack of time (as a consequence of short staffing). Despite this, staff tried to not drop TDRS, and rather an effort was done to fit it in, even if it meant a longer wait for patients:  *When it’s a really, really busy day and it’s just everybody’s busy because the CMAs work well together in helping each other.  If [the MAs] can’t do that before I see the patient… I’m right on top of them then, trying to get that done after. When they’re trying to do… you know… we’re running 3, 4 rooms at a time, then sometimes that can be an issue and the patient has to wait.* [Staff, 103] | |
|  | **CIFR Ratings:** | **-1** | **0** | **+1** | **+1** | **+2** | **+2** |
|  | LEADERSHIP ENGAGEMENT | In one clinic, some leaders were not supportive of TDRS, did not see it as a priority, and would sometimes advocate against it during busier clinics. Overall, there seemed to be an effort to advocate for TDRS from the administrative leadership, who reinforced TDRS during meetings and included it as a quality measure for the organization, but this effort seemed to be less perceived by providers and staff who were mostly un-engaged:    *There’s some providers that say ‘Now you need to go ahead and put them in the room. Now I don’t have time for you to wait and get the retinopathy screening’…*  [Administrator, 104)  In the second site, there seemed to be an effort to advocate for TDRS from the administration, but this effort was less perceived by providers and seemed to translate into a perception of weak leadership. Illustrating this point, one provider felt like the decision to perform TDRS was mostly up to them, and another one referred seldom hearing reinforcements about TDRS:  *In our staff meetings it hasn’t been explicitly talked about very recently that I can remember. It may have been talked about at one of the past meetings […]. I would say, over the past like year and a half […], it’s been brought up […] two or three times. It’s not something that’s often discussed.* (Provider, 104) | | Both clinics showcased relatively strong leadership engagement from administrators, but a relatively low level of engagement by providers (which we consider leaders for TDRS, as they have the clinical ability to determine execution/request the exam):  *Our lead person that comes around tells us also [to perform TDRS] but it mainly comes out in the staff meetings we have once a month* [Staff, 105]  *[The Medical Director] meets with the providers every month and he frequently on the agenda will review what’s happening with the retinal screens. I know he frequently you know, is a real champion himself with the providers encouraging them to get those numbers up.* [Staff, 106] | | Leadership, providers, and lower level supervisors were strongly engaged and committed to TDRS in both clinics. The administrator explained how leadership engaged very frequently with providers and staff and asked for their feedback, ideas for improvement, and reinforced the importance of TDRS for achieving quality measures:  *[We have] bulletin boards in every break room.  It’s also on our intranet.  So there’s lots of data and then our clinical data analyst, one of the things that I asked of her was to take snippets of the conversation from our staff meetings. So, it’s not only like I’m going to put the graph up that somebody could ignore, but I’m going to put this call out feature, ‘great job location X’, and call out successes or call out specific care teams to say, ‘My goodness, you know this is the top in the organization, tell us what you’re doing differently’.  Or on a metric that we’re struggling with, ‘Hey, deposit your idea for improving this metric in this envelope’.*  [Administrator, 101]  Staff felt engaged and heard, and relayed the multiple ways in each leadership engaged in conversations and request for feedback:  *[The leaders] are always asking, you know, ‘What can we do?’. You know, especially if there’s clinics that are lower numbers, ‘If your clinic is better, what are you doing that maybe they’re not doing, what can we do to change this or implement it or make it better?’.  And just trying to get feedback, not just in [staff] meetings but from all the providers in different locations*. (Provider, 103) | |
|  | **CIFR Ratings:** | **-1** | **0** | **+1** | **+1** | **+2** | **+2** |
|  | GOALS AND FEEDBACK | In both clinics, the administration provided some feedback to providers and staff through anecdotal results, but there was no infrastructure for data reporting (graphs, performance statistics) or features for feedback such as quality tabs.  At one site, staff and providers felt like feedback wasn’t provided frequently, and there was a lack of specific numbers/data on TDRS:  *We have monthly meetings but it’s not really brought up, but if there was an issue that would be the place that we would be able to bring that up in* (Staff, 102)  One provider also mentioned:  *We have reports on, I call them maybe the big items, maybe hemoccult screenings and mammograms and what not, but I know it’s her intent to go deeper into it and I’m pretty sure she’s working with IT on [TDRS reports] right now […], it just hasn’t been done yet.* (Provider 102) | | In one clinic, frequent and detailed feedback was provided during monthly and quarterly meetings and in break room boards. However, one staff mentioned the feedback was somewhat vague, as it was given for other diabetic measures, and not specifically for TDRS. A lack of trust on the feedback data was also expressed:  *They’ll say where we’re at along that line and to what they aim for.  So if we’re way down you know they say “You need to get this”, but I don’t trust their numbers too good.  So far they’ve not said too much about the retinopathy but they do on the A1C…* [Staff, 105]  In another clinic, participants mentioned some feedback on goals and performance. Goals at this institution were set and revised periodically by an internal QI committee. This was acknowledged as positive by providers/staff. Additionally, the TDRS champion created a monthly newsletter that contained information on rates of TDRS, and reminders about the availability of the service:  *Usually they just tell us monthly at the staff meetings, that’s the biggest place that I’ve seen it addressed. I actually… go to the quality improvement meeting once a month and so I see it there too, so usually around once a month.* [Provider, 106] | | Both clinics tracked program data and provided feedback based on the data, in monthly meetings and with break room “call out buttons”. Multiple stakeholders seemed to appreciate the effectiveness of this strategy and were aware of their TDRS rates, as well as the organization’s goals:  *We have a bulletin board in our staff room that shows quality measures, so if it’s time for the retinopathy or diabetes it shows up there, the graph and how each doctor is doing.  And then during our staff meeting attended by all employees we see the graph or “this is how we’re doing on our diabetic retinopathy measure”.* [Provider, 101)  *We have monthly staff meetings. They do quality meetings once a month and those qualities are often sent out, especially if there’s a drastic increase, decrease. There’s a quality board in our break room as well that they update about every month or so with different quality measures as far as how we’re doing. And those are done out by site, organization in general, as well as split out by provider as to where you are based on the goal… If you’re really way below the goal then they’ll send me maybe an email or something and say ‘Hey, this is where you’re falling, what can we do? Ideas?’. If you’re exceeding or meeting that goal and a lot of others are struggling then they’ll again ask ‘Hey what are you doing, what can we do?’*  [Staff, 103] | |
| **PROCESS** | |  | |  | |  | |
|  | **CIFR Ratings:** | **-1** | **-1** | **Missing** | **0** | **Missing** | **+2** |
|  | ENGAGING | Both clinics lacked positive opinion leaders, and in one clinic we heard the providers worked as negative opinion leaders:  *I think the focus is more on controlling the hemoglobin A1C, so I think that’s probably is what people look at, at the physician level. And I think what the physician says, kind of flows down to the support staff and everyone around them. (Administrator, 104)*  The absence of education also had a negative effect in both sites:  *They need to feel empowered to make that decision and why they’re doing it. It’s not just another checked box on their list, that “They’re diabetic, I have to do this, I have to do this, what happens if I don’t do this check box?”. So I do think if we had more education for them, for them the support staff, that it may become, it may help us bump that priority side*. [Staff supervisor, 102]  One supervisor mentioned the success of an engaging intervention, via education, for a different screening exam:  *Education for staff why is it important and… I wish it would be… like, for example, [the] cancer society came to me with movies and little pictures, you know, what is normal, what is abnormal. And I truly think it would be extremely helpful to patients for [the] support staff to see that, listen, “this is the normal image, this is not normal image, this is normal image for this age”. […]. I did a pilot of tests on colonoscopies and colon cancer screenings and after I brought [the] cancer society with all their educational session and they ran this program for me for a whole day, my screening numbers went up like crazy.* [Staff supervisor, 104] | | Several participants felt like more reminders and information/education for staff, providers, and patients would be beneficial:  *If there’s any like basic education they could give us. If there’s anything that we could do to help the patient, to either get in faster, to know the importance of what needs to be seen quicker than others, and so we can emphasize it to the patient on how urgent some things are, that might be helpful and beneficial…* [Provider, 106] | | Participants mentioned strategies that the administration used in the initial phase of TDRS implementation to engage providers and staff:  *Especially when [TDRS] started, [the administration was] very involved in the implementation, in the education part, making everybody aware of what was coming, why it was coming, how we were going to do it.  What, always the one typically asking the questions of “What do we need to do to make this better, easier to use, flow better?”, whatever to help.* [Provider, 103]  One provider also gave examples of strategies he frequently used to engage and educate patients on TDRS:  *I try to explain that to patients, that one [diabetic exam] is not really more important than the other.  They’re all equally important and just because you don’t have any vision changes right now doesn’t mean that there’s nothing going on back there that may not be a concern.* [Provider, 103] | |
|  | **CIFR Ratings:** | **-1** | **-1** | **Missing** | **+2** | **+2** | **Missing** |
|  | CHAMPION | Neither site had a champion for TDRS. In one site, one participant mentioned that the previous staff supervisor was very supportive of TDRS in a champion manner, but had been moved to a different position, leaving the initiative without that support. The Administrator acknowledged the potential benefits of a champion, but had a passive attitude towards nominating one. | | All participants noted the presence of a strong champion who championed the intervention in the institution, trained the staff, improved the conditions for the exam, sent out details about TDRS on a monthly letter, and pushed for the implementation of a standing order. The general feeling was that the champion had a major role in the success of TDRS:  *She has a small staff but I, from my understanding, from our discussions at quality improvement meetings that she’s trained them very well to be able to, to perform the screens. And she has a lot of really good oversight, she’s the one that puts the reports together and gets the [TDRS] numbers and I would definitely call her a champion for it* [Provider, 106] | | In one site, participants noted the presence of a champion who pushed for TDRS execution by other providers and teams. The organization set TDRS as a quality measure, and the champion took it upon herself to achieve that quality measure:  *I sort of just like, we are lead providers and there’s a group of us so [TDRS] is, you know, a quality measure, something we push. We want our patients to do this and this is so easy, so convenient.  So for other clinics the other lead providers are also very aggressive in doing [TDRS].* [Provider 101] | |
